# Research on Oracle Technology Based on Multi-Threshold Aggregate Signature Algorithm and Enhanced Trustworthy Oracle Reputation Mechanism

**DOI:** 10.3390/s24020502

**Published:** 2024-01-13

**Authors:** Zhiyuan Wang, Mingan Gao, Gehao Lu

**Affiliations:** School of Information Science & Engineering, Yunnan University, Kunming 650500, China; wzyyx@mail.ynu.edu.cn (Z.W.); gaomingan@stu.ynu.edu.cn (M.G.)

**Keywords:** IoT, sensor data security, blockchain oracle, MTAS signature algorithm, ETROM reputation mechanism, data traceability, data tampering

## Abstract

In the realm of IoT sensor data security, particularly in areas like agricultural product traceability, the challenges of ensuring product origin and quality are paramount. This research presents a novel blockchain oracle solution integrating an enhanced MTAS signature algorithm derived from the Schnorr signature algorithm. The key improvement lies in the automatic adaptation of flexible threshold values based on the current scenario, catering to diverse security and efficiency requirements. Utilizing the continuously increasing block height of the blockchain as a pivotal blinding parameter, our approach strengthens signature verifiability and security. By combining the block height with signature parameters, we devise a distinctive signing scheme reliant on a globally immutable timestamp. Additionally, this study introduces a reliable oracle reputation mechanism for monitoring and assessing oracle node performance, maintaining both local and global reputations. This mechanism leverages smart contracts to evaluate each oracle’s historical service, penalizing or removing nodes engaged in inappropriate behaviors. Experimental results highlight the innovative contributions of our approach to enhancing on-chain efficiency and fortifying security during the on-chain process, offering promising advancements for secure and efficient IoT sensor data transmission.

## 1. Introduction

The inventor of Bitcoin, Satoshi Nakamoto, first introduced the concept of blockchain in 2008, as outlined in his paper “Bitcoin: A Peer-to-Peer Electronic Cash System” [1]. In this paper, Nakamoto meticulously detailed the operational principles of Bitcoin, as well as the foundational principles of blockchain technology. Blockchain, which can be thought of as a distributed ledger technology originally designed for recording transactions of the digital currency Bitcoin, is a decentralized and transparent database that utilizes cryptography to ensure data security. The development of blockchain technology has resulted in significant advancements across various domains. Notably, it has served as the foundation for the emergence of digital currency, including cryptocurrencies such as Bitcoin, and has facilitated innovative developments in digital identity verification, supply chain management, and the Internet of Things (IoT). Crucially, blockchain technology is characterized by its tamper resistance, decentralization, immutability, and traceability, rendering it a monumental technological innovation [2].

Oracles are an essential component in blockchain technology, serving as a kind of middleware for data calling and accessing. They essentially address two key problems, namely, “yes or no” and “what is the data”, thus enabling smart contracts to call external data. Initially, oracles were introduced in the Bitcoin blockchain to fetch exchange rate data [3]. This development can be traced back to the early stages of blockchain technology when it became apparent that a significant limitation was the inability to access external data [4]. As blockchain technology progressed, the scope of oracle applications expanded, and they are now widely used in digital currency exchanges, insurance, financial derivatives, and various other domains. Their primary function is to input real-world data into the blockchain, allowing it to access external data and verify their authenticity. Oracles play a crucial role in digital currency exchanges by providing external digital currency price information for the execution of smart contracts. In the insurance sector, oracles can supply weather and earthquake information to trigger insurance claims. Similarly, in the realm of financial derivatives, oracles provide external data for pricing and settlement purposes [5]. This integration of real-world data into the blockchain facilitates the interaction between the blockchain and the real world.

The integration of blockchain oracle technology into agricultural product traceability in the context of the Internet of Things (IoT) ecosystem is pivotal to enhancing security and traceability. Agricultural product traceability is critical for ensuring food safety and quality [6]. Traditional supply chain systems often face challenges related to transparency, data integrity, and traceability, which pose risks to food safety and quality assurance. Hence, in response to these shortcomings, leveraging blockchain oracle technology along with IoT sensors emerges as a compelling solution. Our approach aims to address these challenges by providing a secure, transparent, and traceable system, thereby mitigating risks associated with data integrity and establishing a foundation for building consumer trust and confidence in the food supply chain. The use of blockchain technology offers a robust solution to these challenges by creating a secure and transparent platform for tracking the journey of agricultural products from farm to table. This application involves the integration of IoT sensors and devices to collect data related to environmental conditions, product handling, and transportation. Subsequently, the data are recorded on the blockchain, resulting in an immutable and auditable record [7].

This paper presents an enhanced blockchain oracle solution founded on the MTAS (Multi-Threshold Aggregate Signature) algorithm and the ETORM (Enhanced Trustworthy Oracle Reputation Mechanism), utilizing the Schnorr threshold aggregate signature [8]. The MTAS signature algorithm introduces the concept of multiple thresholds, necessitating the joint participation of a predetermined number of users to generate a valid signature, as determined by the threshold value. It is worth noting that this dynamic threshold value offers adaptability, allowing customization for each signature process to cater to the application scenario for the secure transmission of IoT sensor data.

The remainder of this manuscript is structured as follows: In Section 2, we provide a comprehensive review of the related work. Section 3 introduces the Materials and Methods used in our research. Section 4 is dedicated to presenting our Experimental Design and Results Analysis. Section 5 covers the Evaluation of our approach. Lastly, in Section 6, we present our Conclusions and outline Future Work.

## 2. Related Work

The choice of oracle technology plays a crucial role in ensuring transparency, security, and reliability within the IoT ecosystem for agricultural product traceability. The two main categories of existing implementations of blockchain oracles are centralized oracles (Oracle) and distributed oracles (Chainlink). Centralized oracles rely on Trusted Execution Environments (TEEs) [9], and their trust models mainly involve voting strategies and reputation-based strategies. These voting strategies determine outcomes based on participants’ interests, while reputation-based strategies depend on the accuracy and integrity of reputation and data authenticity proof mechanisms [10]. This traditional approach has been effective in achieving the goals of providing accurate and trustworthy data related to the origin and journey of agricultural products. However, as the IoT ecosystem expands, distributed oracles, like the Chainlink network, offer an alternative approach that leverages decentralized consensus and multiple data sources to enhance the traceability and transparency of agricultural products [11]. The application of blockchain oracles in this domain aims to address challenges related to data authenticity, integrity, and traceability, ensuring that end consumers can have full confidence in the origin and quality of the products they consume.

Several main implementations of blockchain oracles and signature algorithms have been explored in previous research:

- Provable (formerly known as Oraclize), a centralized oracle service, has been operational since 2015. It relies on auditable virtual machines, such as TLSNotary, and deploys a variety of signature algorithms, including ECDSA (Elliptic Curve Digital Signature Algorithm) [12] and other related signature schemes. These algorithms are vital components in constructing authenticity proofs to ensure the authenticity and integrity of data [13].
- TownCrier is a system that provides secure centralized oracle data transmission services. It utilizes data verification through TLSNotary within a Trusted Execution Environment (TEE). The RSA (Rivest–Shamir–Adleman) signature algorithm plays a crucial role in contributing to data authenticity and integrity verification within this framework [14].
- TLS-N is the first oracle solution that relies on content-extractable signatures, making use of the BLS (Boneh–Lynn–Shacham) signature scheme to generate non-interactive session proofs from blockchain smart contracts. The BLS signature scheme plays a crucial role in TLS-N’s functionality by guaranteeing secure and verifiable data transmission [15].
- The DOS Network is a scalable Layer-2 protocol that employs threshold signatures to achieve consensus among nodes. This consensus mechanism is enabled through the frequent use of the BLS (Boneh–Lynn–Shacham) signature scheme, which ensures secure data transmission and verifiable computation oracles within the network [16].
- Chainlink: Chainlink, operating within a distributed trust model, relies on Chainlink nodes and oracle smart contracts. In the context of data verification, Chainlink often utilizes ECDSA (Elliptic Curve Digital Signature Algorithm) and similar signature schemes to ensure the authenticity and security of data transmitted between smart contracts and web APIs [17].
- Truora, an open-source oracle solution developed by WeBank, is designed for consortium blockchains. It employs the RSA (Rivest–Shamir–Adleman) signature algorithm in both centralized and distributed hybrid modes, playing a pivotal role in providing authenticity proofs for acquired data and enhancing data security and integrity [18]. This implementation is crucial for ensuring the reliability and trustworthiness of the data within consortium blockchains, thereby contributing to the overall security and stability of the system.

Shang Yu et al. proposed a method to improve efficiency by changing the verification algorithm and analyzed the solution to side-channel attacks on ECDSA. However, the current attack methods and the vulnerabilities of ECDSA required lowering its efficiency to enhance security, limiting its application scope. Additionally, ECDSA still could not effectively defend against side-channel attacks [19]. Sober M et al. used BLS threshold aggregate signatures to generate a group private key off-chain and performed group signatures on on-chain data. However, their scheme had great difficulty in signature verification, resulting in higher overall time costs and gas consumption [20]. Liu Wei et al. proposed a prediction machine scheme based on Schnorr threshold aggregate signatures, which had lower computational complexity, storage space overhead, and communication load. However, their scheme did not consider the randomness security of Schnorr signatures and was susceptible to data leaks [21].

Our research is centered on advancing trust and security within blockchain systems, with a specific focus on improving traceability within the Internet of Things (IoT) ecosystem, particularly in the context of agricultural product traceability. Oracles, as critical components in blockchain networks, act as links between blockchain platforms and real-world data sources [22]. The establishment of secure and reliable oracles has posed a significant challenge in this context. While existing research primarily emphasizes highly decentralized scenarios, our work adopts a distinct approach by aiming to comprehensively examine oracle categorization, fundamental methodologies, and tailored design considerations [23]. Consequently, our research outcomes contribute secure and trusted oracle solutions that are well suited for enhancing the traceability of agricultural products within the IoT ecosystem. This, in turn, facilitates the practical adoption of blockchain technology in these fields, ensuring the protection of sensitive data and the validation of data accuracy and bolstering the potential of blockchain technology in the IoT domain.

## 3. Materials and Methods

### 3.1. Implementation of Consortium Blockchain and Chainlink

In order to research the proposed oracle solution, it is imperative to establish a distributed network using the Fabric consortium chain [24]. This network will be built upon a blockchain platform with distributed ledger and smart contract capabilities. Hyperledger Fabric, a widely utilized open-source consortium blockchain technology for enterprise blockchain system development, is deemed to be the most suitable platform for this purpose [25]. The setup of this network will encompass processes such as chaincode development, node configuration, and channel establishment. This will involve defining nodes, channels, and smart contracts to facilitate data storage and management.

The overall architecture of the oracle solution is designed and implemented, encompassing the business system, blockchain, smart contracts, and oracles. The roles in the oracle workflow primarily comprise three components: on-chain user smart contracts (i.e., blockchain smart contracts), on-chain oracle smart contracts, and off-chain external data sources. The workflow for trusted data on-chain involves the following steps.

- User smart contracts initiate data requests to the oracle smart contracts;
- Data are sent to the oracle smart contracts by external data sources;
- The oracle smart contracts return data to the user smart contracts, providing feedback.

**Figure 1:**
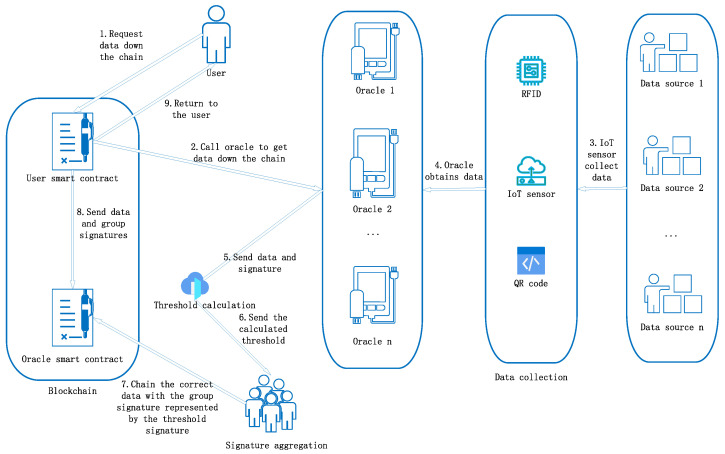
Data flow direction.

The data circulation process in the traceability system is illustrated in Figure 1. It begins with the user smart contract initiating a data request to the oracle smart contract, which, in turn, triggers the retrieval of the data. The Data Acquisition Layer, which incorporates IoT sensors, 2D barcodes, and RFID technology, meticulously captures data related to various stages of the agricultural product. These data are then secured using the MTAS signature algorithm, processed by the oracle smart contract, and finally returned to the user through the user smart contract. The use of blockchain technology ensures robust tracking and accounting, while the involvement of multiple oracle nodes enhances system resilience by collecting data from diverse sources. The visual representation in Figure 1 effectively showcases the dynamic and secure flow of data, highlighting the crucial role played by advanced technologies in the Data Acquisition Layer in the traceability system.

The oracle solution’s transaction process, which enhances the traceability of agricultural products within the IoT ecosystem, entails block generation and transaction creation. Initially, block generation is achieved through a consensus algorithm, which not only serves to ensure transaction consistency and security but also plays a crucial role in safeguarding the traceability of agricultural products. Conversely, users initiate transaction creation through transaction requests, which subsequently undergo verification and authorization before being permanently appended to the blockchain as immutable records. This process ensures the credibility, security, and transparency of the oracle solution for agricultural product traceability.

1. Blockchain Network Setup for Agricultural Product Traceability:
  - In this oracle solution, a distributed network is constructed using Hyperledger Fabric, a consortium blockchain platform.
  - The network encompasses multiple nodes, each maintaining a replica of the ledger data to ensure the comprehensive traceability of agricultural products.
2. Identity Authentication and Authorization:
  - Users of the oracle solution within the agricultural product traceability system authenticate their identities through a mechanism designed to protect sensitive data.
  - Successful authentication leads to the granting of user authorization, specifying their operational scope and permissions regarding agricultural product traceability.
3. Creation of Transaction Requests for Agricultural Product Traceability:
  - Users submit transaction requests through the oracle solution’s business system, focusing on activities related to agricultural product traceability.
  - Transaction requests include detailed information, such as the type of transaction (e.g., data transfer or data querying), the identities of the parties involved, and the transaction amount concerning agricultural products.
4. Broadcasting of Transaction Requests Across the Agricultural Product Traceability Network:
  - Transaction requests are sent to and broadcasted across all nodes in the blockchain network, ensuring the complete traceability of agricultural products.
  - Each node in the network receives transaction requests, adding them to a pending transaction pool, thus facilitating the comprehensive traceability process.
5. Transaction Verification and Execution for Agricultural Product Traceability:
  - Nodes in the blockchain network meticulously verify transaction requests to ensure the legality and validity of agricultural product traceability processes.
  - Verification procedures include thorough checks of digital signatures, validation of the identities of the parties involved, and adherence to predefined rules for transaction amounts within the agricultural product traceability framework.
  - Once verified, nodes execute the transactions and diligently update the ledger data, guaranteeing the precision of agricultural product traceability.
6. Transaction Confirmation and Packaging to Enhance Agricultural Product Traceability:
  - Nodes generate a new block following the successful execution of transactions, packaging the transactions from the pending transaction pool into it.
  - The new block acts as a secure repository for verified and executed transactions, contributing to the accuracy of agricultural product traceability.
7. Utilization of Consensus Mechanisms for Enhanced Agricultural Product Traceability:
  - The agricultural product traceability process relies on consensus mechanisms to validate the generation of new blocks.
  - Common consensus algorithms utilized in this oracle solution include Byzantine Fault Tolerance (BFT) and Proof of Stake (PoS) algorithms [26], ensuring the security of agricultural product traceability.
  - Nodes actively participate in consensus processes to verify the legality and consistency of adding new blocks to the blockchain, specifically concerning agricultural product traceability.
8. Integration of Chainlink Oracle Nodes for Agricultural Product Traceability:
  - Within the blockchain network, Chainlink Oracle nodes play a pivotal role in providing external data to smart contracts, a crucial aspect of agricultural product traceability.
  - When a transaction request necessitates external data, the oracle solution seamlessly interacts with Chainlink Oracle nodes, which fetch data from various off-chain sources, ensuring the accuracy and reliability of data related to agricultural product traceability.
  - The integrated Chainlink nodes ensure that the blockchain network can securely access real-world data, execute transactions based on these data, and maintain a high level of trust within the agricultural product traceability framework.
9. Blockchain Update:
  - Upon reaching a consensus, a new block is appended to the end of the blockchain, securing transaction records permanently within the agricultural product traceability system.

**Figure 2:**
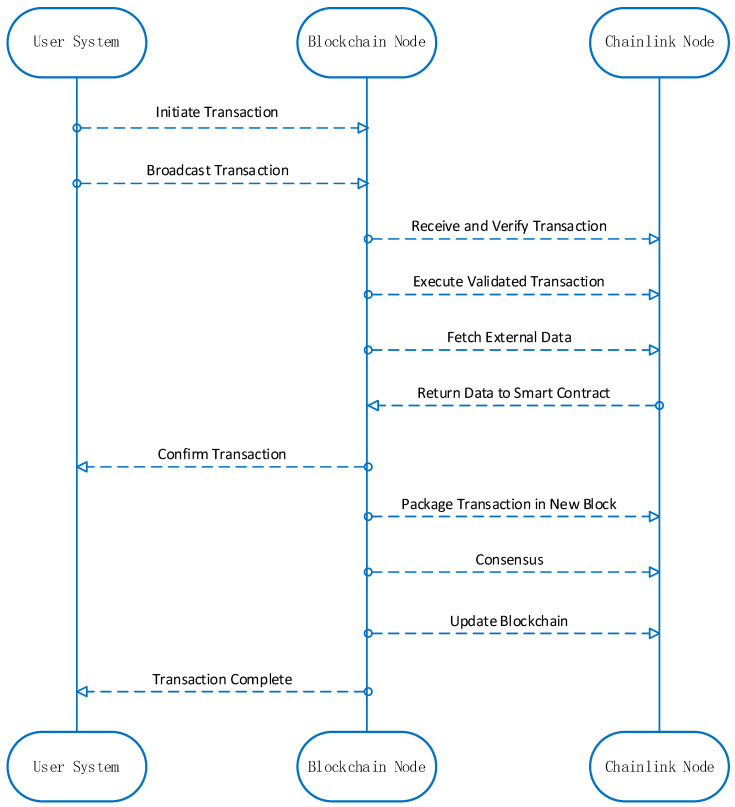
Transaction process.

Figure 2 illustrates the data transaction process used in our experiment, emphasizing the crucial role of the on-chain oracle, Chainlink. This process efficiently manages and verifies data exchange within a consortium blockchain designed to enhance agricultural product traceability in the IoT ecosystem. Identity validation and authorization ensure that users with confirmed identities gain specific operational privileges. Users can submit various transaction requests through our system, which are broadcast to all blockchain nodes.

The on-chain oracle, Chainlink, facilitates the injection of external data, such as exchange rates, weather conditions, and market data, into the blockchain. These data play a critical role in smart contract execution and automated data transfer, as they enable nodes to verify and execute transactions while ensuring digital signature validation and compliance with predefined rules. Following transaction execution, nodes produce a new block, which is subjected to the consensus mechanism to uphold its legitimacy and consistency. This new block is then added to the blockchain, permanently storing transaction records and enhancing the functionality of blockchain applications in bolstering agricultural product traceability within the IoT ecosystem.

### 3.2. Blinding Mechanism Based on Block Height

In this study, we introduce an innovative enhancement to the MTAS algorithm through the incorporation of a novel blind signature mechanism based on blockchain height. Leveraging the continuously increasing block height of the blockchain as a key blinding parameter, our approach enhances the verifiability and security of signatures. By integrating block height with signature parameters, we have devised a distinctive signature scheme that relies more on the globally immutable timestamp. This method not only elevates the credibility of signatures but also enhances their security by requiring attackers to anticipate future block heights for a successful forgery. This improvement injects greater resilience and traceability into the MTAS algorithm, paving the way for advancements in the field of secure communications. The following is a specific introduction:

1. Block Height Parameter Generation: In the new blind signature mechanism, both signers and verifiers generate block height parameters based on the blockchain’s block height. The block height is an ever-increasing value on the blockchain and can be considered an immutable global timestamp.
2. Block Height-Dependent Blinding: During the process of signature generation and verification, block height parameters are used to blind key parameters in the signature process.
3. Signature Generation: Signers use block height parameters to generate blind signatures, ensuring that the signature generation depends on the current blockchain block height. This enhances the verifiability of signatures, as verifiers can check whether the signature is related to a specific block height.
4. Signature Verification: Verifiers need to be aware of the relevant block height when verifying the signatures. The validity of the signature depends on whether the block height parameters match the signature.

### 3.3. Principle and Implementation of MTAS Signature Technology

Here is the basic principle of the MTAS signature algorithm:

1. Public-Key Generation:
  - The system administrator generates a set of public–private-key pairs and publishes the public keys for users participating in the signature process.
  - The public key includes the following parameters: (p,q,g,h). Here, *p* and *q* are two large prime numbers, *g* is a primitive root modulo *p*, and *h* is the exponent of *g*. *x* represents the private key.
    (1) $$h=g^{x}\;\mathrm{mod}\;p$$
  - Signers and verifiers generate the blockHeight parameter, representing the current blockchain block’s height.
2. Signature Generation:
  Assuming there are signers who possess the private keys $x_{1},x_{2},...,x_{n}$, the following steps are performed when a user wants to generate a signature:
  - Choose a random number r and compute $R=g^{r}\;\mathrm{mod}\;p$. This temporary public key *R* is used in the signature process.
  - Calculate the hash value $h^{\prime }=H\left(m\right)$ of the message to be signed, where *H* is a hash function.
  - Compute the blockHeight-dependent blinding parameter:
    (2) $$M_{r}=\left(R+\mathrm{blockHeight}\right)\;\mathrm{mod}\;p$$
  - Based on the current scenario’s requirements, automatically set a flexible threshold value to ensure security and efficiency. Compute it as follows:
    (3) $$t=\frac{\alpha \left(1-\beta \right)\gamma^{2}}{\delta +\xi }$$
    Here, α represents factors related to the application scenario, such as data sensitivity and risk tolerance. We can use a value between 0 and 1 to indicate its weight, with 1 representing the highest weight. β represents factors related to system performance, such as computing resources, network bandwidth, and latency requirements. We can use a value between 0 and 1 to indicate its weight, with 1 representing the highest weight. Here, we use (1−β) to denote the inverse weight of performance factors, meaning that the higher the performance, the lower the weight. γ represents the weight of security indicators, used to measure the importance of security, such as avoiding private-key leakage and resisting attacks. We can use a value between 0 and 1 to indicate its weight, with 1 representing the highest weight. Here, we use $\gamma^{2}$ to denote the quadratic weight of security indicators, emphasizing their importance. δ represents the weight of efficiency indicators, used to measure the importance of efficiency, such as computational and communication overhead limitations. We can use a value between 0 and 1 to indicate its weight, with 1 representing the highest weight. ξ represents the adjustment parameter used to balance security and efficiency. It can be adjusted based on actual requirements. For example, increasing the value of ξ can reduce the threshold value, thereby improving efficiency but potentially decreasing security.
  - If the number of signers participating in the signature reaches or exceeds the threshold value *t*, perform the signature calculation for $h^{\prime }$. Use $M_{r}$ instead of *R* in the signature calculation:
    (4) $$s=Sign(x,h^{\prime },M_{r})$$
    where Sign is the signature algorithm.
  - Sum up the signatures that satisfy the threshold condition:
    (5) $$S=\sum_{1}^{n}s_{i}$$
3. Signature Verification:
  When receiving a signature message (S,R,blockHeight), the verifier needs to perform the following steps for verification:
  - First, verify whether *R* is a valid public key by checking whether it satisfies the conditions during the public-key generation phase.
  - Calculate the hash value $h^{\prime }=H\left(m\right)$ of the message to be verified, ensuring that the hash value used in the verification process is consistent with the one generated during the signature generation phase.
  - Recalculate the blockHeight-dependent blinding parameter $M_{r}$ using the received blockHeight.
  - Based on the threshold value set for the current scenario, compute the verification formula:
    (6) $$g^{s}\equiv \left(h^{\prime t}{\left(h^{\prime M_{r}}\right)}^{t}\right)\;\mathrm{mod}\;p$$
    where *g* and *p* are parameters from the public key, and *t* is the threshold value.
  - For each signer i(i=1,2,…,n) participating in the signature, compute the verification formula:
    (7) $${\left(h^{\prime R}\right)}^{t}\equiv \left(g^{R_{i}}S_{i}\right)\;\mathrm{mod}\;p$$
    where $R_{i}$ is the temporary public key of signer *i*, and $S_{i}$ is the signature result of signer *i*.
  - If verification formulas (6) and (7) hold true for all signers *i*, the signature is considered verified.

The MTAS signature algorithm achieves security and efficiency requirements in different scenarios by automatically setting a flexible threshold value. This approach enables the selection of appropriate threshold values in different scenarios, satisfying security requirements while maintaining efficiency, thereby ensuring a trustworthy signature generation and verification process [27]. The flexible adjustment of the threshold value allows for dynamic tuning based on factors such as the number of signers, security requirements, and computing resources, ensuring the security and effectiveness of the signature [28].

Furthermore, blockHeight-dependent blinding enhances the verifiability of the signature, as it relies on a global timestamp (blockHeight), which is difficult to forge. It increases the security of the signature, as attackers would need to know the future blockHeight values to forge a valid signature.

Algorithm 1 is aimed at overcoming the security and performance issues in traditional single-signer schemes. It is a public-key cryptography-based digital signature algorithm. In order to enhance the security of the signature, the algorithm involves the participation of multiple signers, requires multiple threshold conditions to be met to complete the signature process, and uses a blinding mechanism based on block height to enhance the verifiability and security of the signature.
**Algorithm 1** MTAS Signature Algorithm1:**procedure** KeyGeneration2:    Generate key pair (pk,sk)3:    Set public-key parameters: (p,q,g,h,x,blockHeight)4:    p,q are large prime numbers5:    *g* is a primitive root modulo *p*6:    $h=g^{x}\;\mathrm{mod}\;p$, where *x* is the private key7:    **return** public key pk8:**end procedure**9:**procedure** SignatureGeneration(messagem)10:    Let *N* be the number of signers11:    Let S={}12:    **for** i=1 to *N* **do**13:        Choose a random number *r*14:        Compute temporary public key $R=g^{r}\;\mathrm{mod}\;p$15:        Calculate the hash value $h^{\prime }=H\left(m\right)$16:        Compute the blockHeight-dependent blinding parameter:17:             $M_{r}=\left(R+\mathrm{blockHeight}\right)\;\mathrm{mod}\;p$18:        Set the flexible threshold value *t* based on the scenario: $t=\frac{\alpha \left(1-\beta \right)\gamma^{2}}{\delta +\xi }$19:        **if** number of signers ≥t **then**20:           Compute the signature: $s_{i}=\mathrm{Sign}\left(x,h^{\prime },M_{r}\right)$21:           Add $s_{i}$ to *S*22:        **end if**23:    **end for**24:    **if** number of signers ≥t **then**25:        **return** valid signature *S*26:    **else**27:        **return** invalid signature28:    **end if**29:**end procedure**30:**procedure** SignatureVerification(messagem,signature(S,R))31:    Verify whether *R* is a valid public key32:    Calculate the hash value $h^{\prime }=H\left(m\right)$33:    Recalculate the blockHeight-dependent blinding parameter $M_{r}$ using the received blockHeight:34:         $M_{r}=\left(R+\mathrm{blockHeight}\right)\;\mathrm{mod}\;p$35:    Based on the threshold value *t*, compute the verification formula: $g^{s}\equiv \left(h^{\prime t}{\left(h^{\prime M_{r}}\right)}^{t}\right)\;\mathrm{mod}\;p$36:    **for** each signer *i* participating in the signature **do**37:        Compute the verification formula: ${\left(h^{\prime R}\right)}^{t}\equiv \left(g^{R_{i}}S_{i}\right)\;\mathrm{mod}\;p$38:        **if** the formula is invalid **then**39:           **return** invalid signature40:        **end if**41:    **end for**42:    **return** valid signature43:**end procedure**

### 3.4. Security Proof for MTAS Signature Scheme with BlockHeight-Based Blinding

#### 3.4.1. Security Assumptions

1. Discrete Logarithm Assumption: The security relies on the computational infeasibility of solving discrete logarithm problems in the finite field defined by large prime numbers *p* and *q*.
2. Primitive Root Assumption: The selection of *g* as a primitive root modulo *p* ensures the difficulty of the discrete logarithm problem.
3. BlockHeight Security: We assume the secure generation and utilization of the blockHeight parameter, considering it a trusted and tamper-resistant global timestamp.
4. Hash Function Security: The hash function *H* is assumed to be collision-resistant and preimage-resistant.
5. Randomness Assumption: The randomness introduced by the selection of random numbers is assumed to be uniformly distributed and unpredictable.

#### 3.4.2. ECDSA Preliminaries and Hardness Assumptions

Before delving into the security analysis of the MTAS algorithm, it is crucial to establish the foundational concepts of the Elliptic Curve Digital Signature Algorithm (ECDSA) and articulate the hardness assumptions that underpin its security.

1. ECDSA Preliminaries: ECDSA is a widely used digital signature algorithm that operates on elliptic curves over finite fields. In ECDSA, key pairs are generated based on the difficulty of solving the elliptic curve discrete logarithm problem. The security of ECDSA relies on the computational infeasibility of deriving a private key from its corresponding public key.
2. Elliptic Curve Discrete Logarithm Problem: The elliptic curve discrete logarithm problem involves finding the exponent *x* in the equation Q=xP for a given base point *P* and a point *Q* on the elliptic curve. This problem is believed to be computationally hard, forming the basis for the security of ECDSA.
3. Key Generation Security: In the MTAS, the security of key generation is directly linked to the assumed hardness of the elliptic curve discrete logarithm problem. The generation of the public key *y* from the private key *x* is secure because deriving *x* from *y* necessitates solving the elliptic curve discrete logarithm problem, which is considered infeasible within the chosen elliptic curve.
4. Signature Generation Security: The MTAS algorithm enhances signature generation security by introducing a blockHeight-based blinding parameter $M_{r}$. The security analysis of signature generation incorporates the computational infeasibility of solving the elliptic curve discrete logarithm problem, particularly with the added complexity introduced by the blockHeight.
5. Signature Verification Security: The security of signature verification in the MTAS relies on the robustness of ECDSA. The verification process involves confirming the validity of the public key, recalculating the blockHeight-dependent blinding parameter $M_{r}$, and applying verification formulas. The inherent hardness of the elliptic curve discrete logarithm problem ensures the integrity of the verification process.

#### 3.4.3. Security Proof Overview

The core enhancement in the MTAS algorithm involves the incorporation of blockHeight-based blinding to address the potential insecurity arising from the random threshold value *t*. The proof is structured as follows:

1. Key Generation Security:
  The security of key generation remains based on the assumed hardness of the discrete logarithm problem. Given $y\equiv g^{x}\;\left(\mathrm{mod}\;p\right)$, an adversary attempting to derive *x* from *y* would need to solve the discrete logarithm problem, which is considered computationally infeasible in the defined finite field.
2. Signature Generation Security:
  The blockHeight-dependent blinding parameter $M_{r}$ is introduced to tie each signature to a specific blockHeight, mitigating the potential insecurity associated with the random threshold value *t*. The security of the signature generation process is contingent upon the computational infeasibility of solving the discrete logarithm problem with the added complexity introduced by the blockHeight.
  Let *S* be the signature set and *t* be the threshold value. For a valid signature, the following condition must hold:
  (8) Numberofsigners≥t
  The probability of an adversary producing a valid signature without knowledge of the private key *x* is negligible due to the inherent security properties of the discrete logarithm problem.
  - Security of $M_{r}$: The security of the blockHeight-dependent blinding parameter $M_{r}$ is crucial. Its computation involves the temporary public key *R* and the blockHeight, introducing additional complexity to the discrete logarithm problem.
3. Signature Verification Security:
  The verification process involves confirming the validity of the public key, recalculating the blockHeight-dependent blinding parameter $M_{r}$, and applying verification formulas. The security of the verification process relies on the difficulty of deriving a valid signature without the private key.
  The verification formula is
  (9) $$g^{S}\equiv \left(h^{\prime t}{\left(h^{\prime M_{r}}\right)}^{t}\right)\;\left(\mathrm{mod}\;p\right)$$
  where *g*, *p*, *t*, $h^{\prime }$, and $M_{r}$ are parameters from the public key.
  For each signer *i* with a temporary public key $R_{i}$ and signature $S_{i}$, the verification formula is
  (10) $${\left(h^{\prime R_{i}}\right)}^{t}\equiv \left(g^{R_{i}}S_{i}\right)\;\left(\mathrm{mod}\;p\right)$$
  If an adversary attempts to produce a signature without the private key, the verification equations are unlikely to hold for a sufficient number of signers due to the computational infeasibility of solving the discrete logarithm problem.

#### 3.4.4. Formal Proof

We integrate the enhanced MTAS algorithm with the blockHeight-based blinding mechanism into the security framework, considering the added complexity introduced by the blockHeight parameter. The SignatureGeneration phase of the MTAS algorithm involves the following steps:

1. Choose a random number *r*.
2. Compute $R=g^{r}\;\mathrm{mod}\;p$.
3. Calculate the hash value $h^{\prime }=H\left(m\right)$.
4. Compute the blockHeight-dependent blinding parameter: $M_{r}=\left(R+\mathrm{blockHeight}\right)\;\mathrm{mod}\;p$.
5. Based on the current scenario’s requirements, automatically set a flexible threshold value *t* to ensure security and efficiency.
6. If the number of signers participating in the signature reaches or exceeds the threshold value *t*, use $M_{r}$ instead of *R* to calculate the signature: $s=\mathrm{Sign}(x,h^{\prime },M_{r})$.

Now, we will prove that the generated signature *s* satisfies the verification equation:

(11) $$g^{s}\equiv \left(h^{\prime t}{\left(h^{\prime M_{r}}\right)}^{t}\right)\;\mathrm{mod}\;p$$

**Proof.**  We will show that the signature *s* satisfies the verification equation.

Starting with the calculation of ${\left(h^{\prime M_{r}}\right)}^{t}$:

$$\begin{array}{cc} {\left(h^{\prime M_{r}}\right)}^{t} & ={\left(h^{\prime (R+\mathrm{blockHeight})\;\mathrm{mod}\;p}\right)}^{t}\\ \; & ={\left(h^{\prime R}\cdot h^{\prime \mathrm{blockHeight}}\right)}^{t}\;(\mathrm{by}\;\mathrm{congruence}\;\mathrm{properties})\\ \; & ={\left(g^{rx}\cdot g^{\mathrm{blockHeight}x}\right)}^{t}\;(\mathrm{by}\;\mathrm{definition},\;h^{\prime }=g^{x})\\ \; & =g^{(r+\mathrm{blockHeight})xt}\;(\mathrm{combining}\;\mathrm{exponent}\;\mathrm{laws}) \end{array}$$

Rewriting the verification equation:

$$\begin{array}{cc} g^{s} & \equiv (h^{\prime t}{\left(h^{\prime M_{r}}\right)}^{t})\;\mathrm{mod}\;p\\ \; & \equiv {\left(g^{xt}\right)}^{t}\cdot g^{(r+\mathrm{blockHeight})xt}\;\mathrm{mod}\;p\;(\mathrm{substituting}\;h^{\prime }=g^{x}\;\mathrm{and}\;\mathrm{the}\;\mathrm{calculated}\;{\left(h^{\prime M_{r}}\right)}^{t})\\ \; & \equiv g^{txt}\cdot g^{(r+\mathrm{blockHeight})xt}\;\mathrm{mod}\;p\;(\mathrm{combining}\;\mathrm{exponent}\;\mathrm{laws})\\ \; & \equiv g^{txt+(r+\mathrm{blockHeight})xt}\;\mathrm{mod}\;p\;(\mathrm{merging}\;\mathrm{exponent}\;\mathrm{terms})\\ \; & \equiv g^{(t+r+\mathrm{blockHeight})xt}\;\mathrm{mod}\;p\;(\mathrm{rearranging}\;\mathrm{exponent}\;\mathrm{terms}) \end{array}$$

This expression matches the exponent in the signature calculation $s=\mathrm{Sign}(x,h^{\prime },M_{r})$. Thus, we have demonstrated that the generated signature *s* satisfies the verification equation.

Therefore, the SignatureGeneration phase adheres to the design properties of the MTAS algorithm.   □

#### 3.4.5. Proof of Correctness

1. Assumptions
  We assume the underlying security of the MTAS signature algorithm based on the discrete logarithm problem.
2. Key Generation
  Let *x* be the private key and *y* be the corresponding public key, calculated as follows:
  (12) $$y\equiv g^{x}\;\left(\mathrm{mod}\;p\right)$$
3. Signature Generation
  When generating a signature for a message *M*, Alice chooses a random *k* and computes the intermediate value *K*:
  (13) $$K\equiv g^{k}\;\left(\mathrm{mod}\;p\right)$$
  The hash value *e* of the message is computed as e=H(M,K). The signature *s* is then calculated:
  (14) s≡k−x·e(modq)
4. Signature Verification
  Upon receiving the message *M* and the signature (s,K), Bob verifies the signature using the public key *y*. Bob checks whether *y* is a valid public key and then computes the hash value *e*:
  (15) e=H(M,K)
  Bob calculates the temporary values $v_{1}$ and $v_{2}$:
  (16) $$v_{1}\equiv g^{s}\;\left(\mathrm{mod}\;p\right)$$
  (17) $$v_{2}\equiv y^{e}\;\left(\mathrm{mod}\;p\right)$$
  Finally, Bob checks whether $v_{1}\equiv v_{2}\;\left(\mathrm{mod}\;p\right)$. If this equation holds, the signature is considered valid.

#### 3.4.6. Advantages of MTAS with Block-Height-Based Blinding Mechanism

The MTAS signature algorithm, enhanced by the introduction of a block-height-based blinding mechanism, offers several notable advantages:

1. Enhanced Security: The incorporation of a block-height-dependent blinding parameter adds an additional layer of security to the signature generation process. By leveraging the continuously increasing and tamper-resistant nature of blockchain block heights, the algorithm strengthens its resistance to certain types of attacks, such as replay attacks and signature forgery.
2. Temporal Dependency: The blinding parameter, determined by the current block height, introduces temporal dependency into signature generation. This means that each signature is uniquely tied to a specific point in time, contributing to the prevention of signature reuse and providing a level of temporal authenticity.
3. Protection Against Threshold Randomization: In the MTAS Algorithm 1 proposed above, the random threshold value *t* introduces an element of randomness that could potentially be exploited. The block-height-based blinding mechanism mitigates this randomness by tying the threshold to a deterministic and publicly verifiable parameter, namely, the block height.
4. Blockchain Integration: Leveraging the block height from a blockchain enhances the algorithm’s integration with blockchain-based systems. This integration aligns with the underlying principles of transparency and immutability associated with blockchain technology.

### 3.5. Reputation Mechanism for Oracle Nodes

#### 3.5.1. The Principle of ETORM

In order to enhance the trustworthiness of the oracle system, the Enhanced Trustworthy Oracle Reputation Mechanism (ETORM) implements a reputation contract. This reputation contract is used to track the historical service performance of each oracle in decentralized oracle systems, which are composed of multiple oracles forming a service network [29]. The purpose of tracking the historical service performance is to identify improper behavior, including free-riding by copying hash values from other oracles, mirror attacks, and Sybil attacks [30]. Oracles involved in such inappropriate behavior have reputation scores and pledged stakes deducted by the reputation contract.

**Figure 3:**
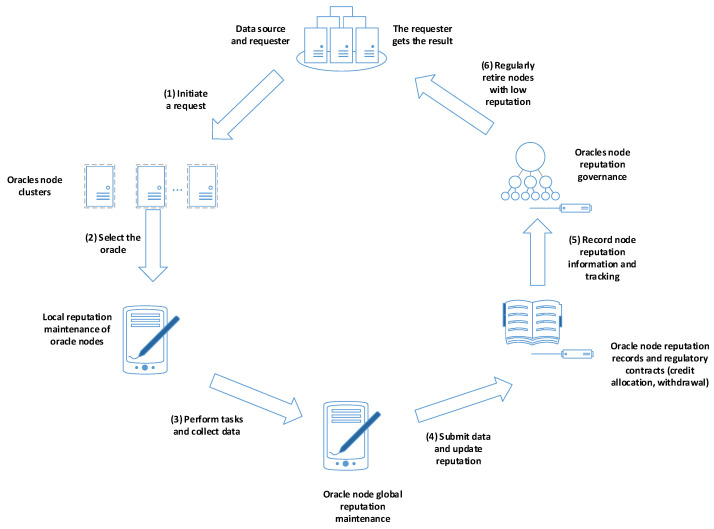
Enhanced trustworthy oracle reputation mechanism.

As shown in Figure 3, the requester and data source initiate a request and send it to the oracle cluster to complete the task requirements. The oracle cluster selects oracle nodes from its pool and assigns the task to the chosen nodes. Each oracle node maintains its local reputation and proceeds to execute the task and collect data. After executing the task, the oracle nodes submit the collected data to the oracle cluster and update their global reputation. To keep track of the reputation information and performance of all oracle nodes, a reputation record and governance contract are responsible for recording and allocating resources. Periodically, the reputation governance mechanism removes oracle nodes with low reputation. The requester retrieves the results submitted by the oracle nodes, while the reputation governance of the oracle nodes ensures the stability and trustworthiness of the oracle cluster by regularly removing nodes with low reputation.

Algorithm 2 introduces a reputation mechanism for each oracle node. This mechanism encompasses both local and global reputation metrics, tailored to enhance the credibility and reliability of traceability data. Local reputation pertains to the completion status of tasks managed by individual nodes. It includes detailed information such as task completion times and the accuracy of results. These data contribute to the assessment of each node’s reliability and performance.

Global reputation, in contrast, evaluates the overall reputation score of each node within the oracle cluster. This holistic score considers various factors, including the node’s historical task completion record, availability, and overall performance. The smart contracts governing the system meticulously record and manage these reputation metrics. When the oracle solution necessitates data from the oracles, the system leverages the global and local reputations of the nodes. This allows for the filtering and ranking of nodes based on their reputation scores, prioritizing those with higher reliability.
**Algorithm 2** ETORM Reputation Mechanism Algorithm**Require:** requester,oracleCluster1:Initialize reputation for each oracleNode in oracleCluster2:**function** ExecuteTaskAndCollectData(oracleNode,task)3:    Execute task and collect data4:    Submit collected data to oracleCluster5:**end function**6:**procedure** RequestAndProcess(dataRequest)7:    task←GenerateTask(dataRequest)8:    selectedNodes← SelectOracleNodes(oracleCluster,len(task))9:    **for** *i* in range(len(task)) **do**10:        oracleNode←selectedNodes[i]11:        ExecuteTaskAndCollectData(oracleNode,task[i])12:    **end for**13:    submittedData← GatherSubmittedData(oracleCluster)14:    **for** oracleNode in oracleCluster **do**15:        UpdateReputation(oracleNode,submittedData)16:        **if** oracleNode.globalReputation<reputationThreshold **then**17:    Remove oracleNode from oracleCluster18:        **end if**19:    **end for**20:**end procedure**

#### 3.5.2. Formal Proof

**Lemma** **1.** *ExecuteTaskAndCollectData* *Correctness*.

**Proof.** 

1. According to the precondition, oracleNode is a valid node in oracleCluster, and task is a valid task.
2. Call ExecuteTaskAndCollectData, which executes the task and collects data.
3. According to the postcondition, oracleNode is still in oracleCluster, and its data have been updated with the result of executing the task.

□

**Lemma** **2.** *UpdateReputation* *Correctness*.

**Proof.** 

1. According to the precondition, oracleNode is a valid node in oracleCluster, and submittedData is valid.
2. Call UpdateReputation, which updates oracleNode’s reputation based on the submitted data.
3. According to the postcondition, oracleNode is still in oracleCluster, and its reputation has been updated based on the submitted data.

□

## 4. Experimental Design and Results Analysis

### 4.1. Experimental Environment

This paper presents the testing and analysis of the MTAS signature algorithm. Conduct experiments as shown in Table 1, compile smart contracts on the locally deployed Fabric network, and use Fabric Go SDK to automatically execute deployment [31]. This research focuses on three main aspects. Firstly, it evaluates the feasibility and efficiency of the MTAS signature algorithm in practical applications, particularly in terms of latency and performance. Secondly, it analyzes the effectiveness of the enhanced trustworthy oracle reputation mechanism in oracle data governance and explores how this mechanism can be used to ensure the credibility and security of oracle data. Lastly, this paper presents tests on the throughput of the proposed oracle data transmission scheme. The following table presents the experimental setup, including the software and hardware components used.

### 4.2. Comparison of Time Consumption of Each Signature Scheme

We will evaluate the latency performance of the MTAS signature algorithm in this oracle solution. By recording the time required for the algorithm to generate and verify signatures in this oracle solution, we can assess the latency during data authentication and transfer requests.

**Figure 4:**
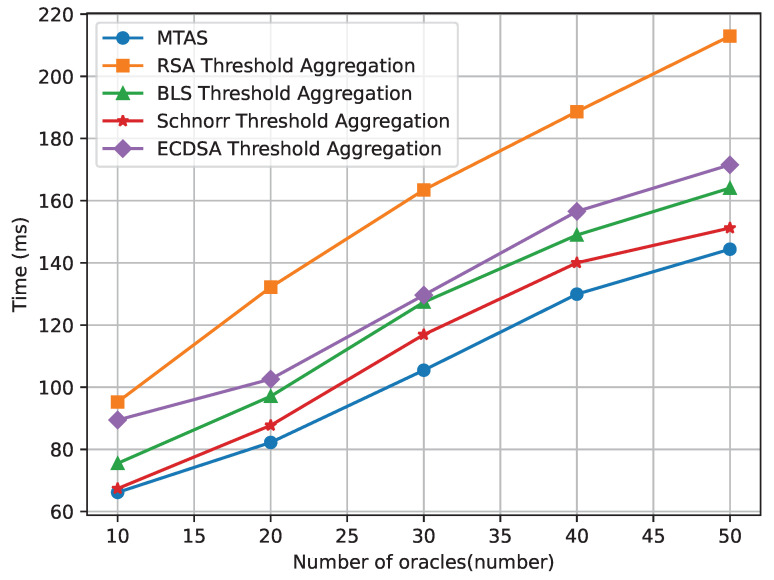
Comparison of time consumption of each signature scheme.

It can be seen in Figure 4 that as the threshold value increases, the required number of participating signers also increases. This leads to a higher level of security and consensus among the signers, ensuring that multiple trusted entities are involved in the signature generation process. Consequently, the time required for signature generation and verification may also increase slightly. This is because more signers need to perform their individual computations and contribute to the aggregate signature, resulting in a longer overall processing time. Comparing the MTAS signature algorithm with other algorithms, such as BLS Threshold Aggregation, ECDSA Threshold Aggregation, Schnorr Threshold Aggregation, and RSA Threshold Aggregation, we can find that the MTAS algorithm leverages the threshold mechanism effectively, balancing security and efficiency. It provides a trade-off between the number of participating signers and the overall processing time, achieving a good balance between trustworthiness and performance. The MTAS signature algorithm exhibits good scalability, allowing the system to handle a larger number of signers and accommodate growing demands for data authentication and transfer. As the threshold value increases, the system can scale up by involving more signers.

### 4.3. Comparison of GAS Consumption of Each Signature Scheme

The data obtained through smart contracts exhibit platform independence, as they can be executed across various systems with a single compilation. While computer scheduling may introduce some minor discrepancies, the overall gas consumption remains relatively constant. Given that on-chain storage operations incur high gas costs, eliminating unnecessary on-chain storage operations is essential. To detect malicious oracle nodes and malicious key distributors, the oracle smart contract must handle requests and messages. To minimize gas consumption, when the oracle smart contract sends requests to the oracle collective or when oracles send messages to the smart contract, these interactions are executed in the form of events rather than being stored in persistent storage. The oracle nodes or the oracle smart contract only needs to read the requests. Additionally, the smart contract employs uint256 and bytes32 variables to store information, reducing the extra costs incurred by type conversion during the Ethereum Virtual Machine runtime.

**Figure 5:**
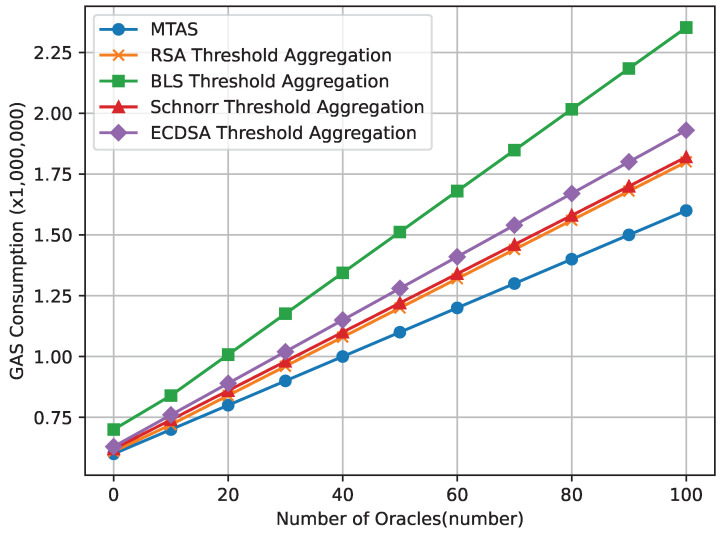
Comparison of GAS consumption of each signature scheme.

As can be seen in Figure 5, the GAS consumption of this scheme is less, and when the number of oracles increases, the GAS consumption of this scheme increases more slowly. In addition, the GAS consumption of all signature schemes does not exceed 2,500,000 wei. The main expense is the storage of the data of agricultural products on the chain, which can effectively achieve the trustworthy uploading of agricultural product data to the chain.

### 4.4. Reputation Mechanism Evaluation

Invoking the reputation contract is crucial for examining the historical performance of oracles to select suitable oracles for service requesters. The selection of oracles is then executed based on specified attributes within the service request, including data sources, response time, and specific data requirements [32]. After the fulfillment of a service request, the Chainlink network records the completion, generates a data report, and adjusts the reputation score of the serving oracle by invoking the reputation contract.

Through simulation experiments, the reputation mechanism of oracle nodes is rigorously tested, employing scores to quantify the magnitudes of local reputation values and global reputation. A comparative assessment of the reputations among multiple oracles can be conducted by calculating the weighted average of the local reputation values. The service requesters undertake the task of accrediting different oracles, and the envisaged criteria encompass the following aspects:

- The total number of accepted requests by the oracle, comprising both executed and pending requests;
- The number of completed requests, which is used to calculate completion-rate bonus points;
- Bonus points, which are determined by counting the total number of requests and are used to judge whether the contract is acceptable compared to the other oracles’ responses, generally measured as the average of the total number of accepted tasks and the total number of completed tasks;
- Average response-time-score reduction;
- The cumulative number of punishment points, where punishment is due to the improper behavior of the prophet (copying the answers or disclosing or using the service request data), and pledges are deducted from the LINK.

**Figure 6:**
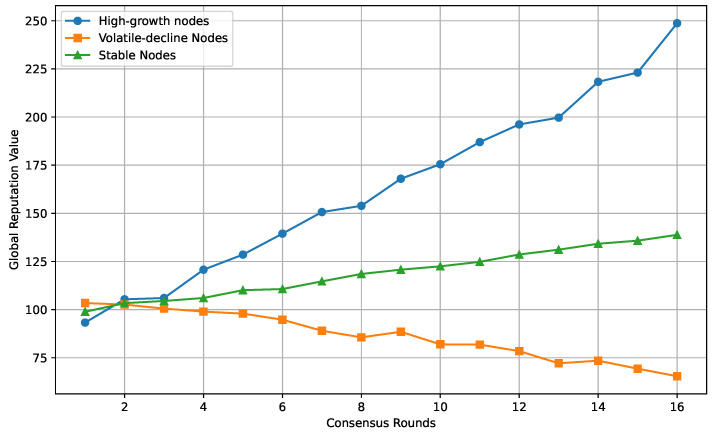
Changes in the global reputation mechanism of oracle nodes.

The oracle nodes are categorized into three types: high-growth nodes, volatile-decline nodes, and stable nodes. As observed in Figure 6, high-growth nodes exhibit remarkable performance within a certain consensus cycle, leading to a rapid increase in the local reputation value. They can potentially stand out as top performers in a given cycle. Volatile-decline nodes experience fluctuations and declining trends in their local reputation values during consensus cycles, possibly due to varying performance across different cycles. Stable nodes consistently maintain their performance throughout consensus cycles, gradually increasing their local reputation values or maintaining them at higher levels. These nodes embody high-reputation entities within the system, consistently demonstrating reliability and consistency.

### 4.5. Agricultural Product Traceability Scenarios

In this part, we present experiments conducted using specific scenarios of traceability of agricultural products in the Internet of Things ecosystem. From the origin, processing, packaging, circulation, and finally, to retail, the entire process is recorded in traceability units through the blockchain.

**Figure 7:**
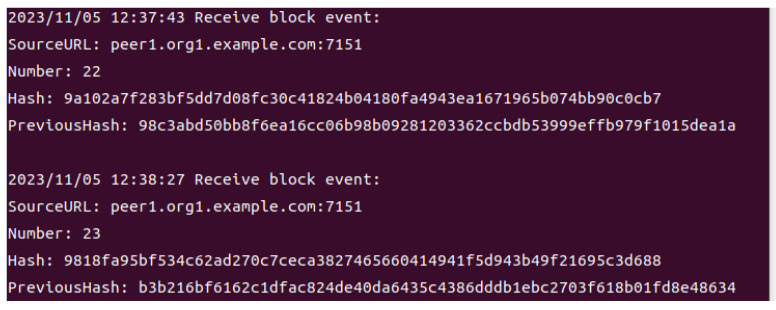
Smart contract event received.

As can be seen in Figure 7, every time a transaction is received, the blockchain system will automatically receive the smart contract event, showing the Source URL, the height of the block, the hash of the block, and the hash of the previous block.

**Figure 8:**
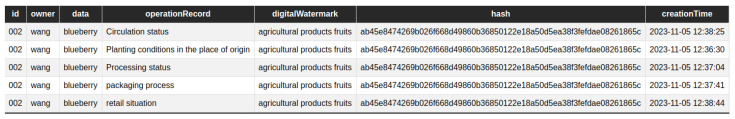
Agricultural product traceability records.

In Figure 8, we can see that every aspect of the traceability record of agricultural products is documented on the blockchain and cannot be tampered with, effectively ensuring the feasibility and security of traceability of agricultural products in the IoT ecosystem.

### 4.6. Throughput Evaluation

To evaluate the efficiency of the proposed solution in the context of agricultural product traceability within the IoT ecosystem, we conducted throughput testing using Tape, a performance assessment tool integrated into Hyperledger Fabric. The Tape tool encompasses two essential components: a load generator client and an observer client; gRPC is utilized for seamless transaction transmission. We further enhanced the testing environment by segmenting transaction and block processing into discrete stages, leveraging coroutines and channel buffering to facilitate parallel processing. This optimization proved invaluable in assessing Fabric’s real-world performance accurately. When evaluating the solution’s efficiency in the context of agricultural product traceability, we observed distinct behavior based on the type of operation. For update operations, the system throughput exhibited a gradual increase with concurrent submission volume, peaking at 697.706 transactions per second (tps) when the transaction volume reached 400. This behavior is attributed to the broader network impact of update operations, which resulted in slightly lower performance compared to query operations.

**Figure 9:**
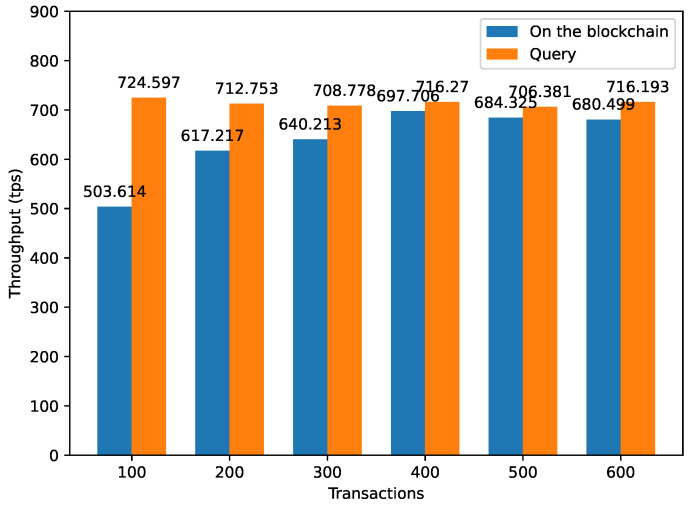
Throughput evaluation.

In contrast, query operations, which primarily involve a single peer node, demonstrated optimal performance at a transaction volume of 100, achieving a remarkable system throughput of 724.597 tps. These findings are exemplified in Figure 9, illustrating the system throughput for both update and query operations, highlighting the practical application of the solution in enhancing agricultural product traceability within the IoT ecosystem.

We conducted throughput tests to evaluate on-chain data submission and query operations, as demonstrated in the graph below:

## 5. Evaluation

The MTAS signature algorithm introduces a versatile approach to ensuring signature security and trustworthiness while accommodating variable threshold values. By allowing for threshold adjustments, the algorithm empowers users to strike the right balance between security and scalability. Higher thresholds offer heightened security by necessitating the involvement of more signers, while lower thresholds promote efficiency and scalability. The dynamic threshold feature provides adaptability to diverse security and efficiency requirements, thus guaranteeing the integrity of signatures. In tandem with the ETORM reputation system, our contribution further introduces a comprehensive mechanism for quantifying local oracle trustworthiness. This innovation enables precise assessments of credibility at the local level, paving the way for informed oracle selection. It represents a pioneering stride in reinforcing trust within decentralized oracles, thereby enhancing trust and reliability in data transmission. Consequently, this dual approach fortifies data integrity and sustains the crucial balance between efficiency and security.

The combined advantages of the ETORM reputation mechanism and the adoption of the MTAS signature algorithm provide a unique and powerful solution in the field of data transmission, making it particularly suitable for managing sensitive information and mission-critical applications within the IoT field. As such, they make a significant contribution to advancing IoT and sensor technology, injecting confidence and promoting greater utility in the field of agricultural product traceability.

## 6. Conclusions and Future Works

The proposed oracle solution, which leverages the MTAS signature algorithm and the ETORM reputation mechanism, holds significant practical value within the Internet of Things (IoT) and sensor domains. Specifically tailored to enhance data security and trustworthiness, the MTAS signature algorithm implements multiple-threshold signature technology, enabling the participation of multiple entities in the signing process. This facet becomes particularly pivotal in IoT and sensor applications, characterized by extensive data exchange and sharing, where the preservation of data integrity and confidentiality stands as a paramount concern [33]. Furthermore, the integration of the ETORM reputation mechanism serves as a critical enabler for safeguarding data credibility through the assessment and vigilance of both local and global reputations of oracle nodes. In IoT environments, ensuring that data originate from a trustworthy source is imperative to avert data tampering or falsification. Therefore, the ETORM reputation mechanism lays a robust trust foundation for IoT and sensor applications.

It is worth noting that a potential limitation of the oracle solution is its reliance on randomness. While randomness introduces essential security features, it may also introduce an element of unpredictability. Addressing this reliance on randomness can potentially enhance predictability and stability in data transmission, which is particularly vital in critical IoT applications. Moreover, ongoing efforts are necessary to adapt the solution to evolving security threats, ensuring it remains resilient to emerging attack vectors.

For future research, there exist opportunities for the further exploration and enhancement of this solution to cater to the burgeoning demand within the realm of IoT and sensor applications. Potential areas for improvement include optimizing the solution’s performance and scalability to meet the requirements of large-scale, high-throughput data scenarios. Expanding the solution’s application scope to broader domains, such as smart health monitoring, environmental protection, and agricultural automation, holds promise. Future endeavors should also center on refining trusted oracle reputation mechanisms to elevate the precision and security of reputation assessments. Ultimately, driving innovation and advancement in the IoT and sensor domains can be attained by exploring and enhancing other blockchain oracle-related technologies, contributing to the continued growth and evolution of this dynamic field.

## Figures and Tables

**Table 1:** Experimental equipment configuration table.

Equipment	Parameter
CPU	11th Gen Intel(R) Core(TM) i5-11400H @ 2.70 GHz
Memory	16 GB
Hard disk	SSD 1 TB
Operating system	Ubuntu 22.04
Golang version	go 1.18.5
Solidity version	solidity 0.7.6
Chainlink version	chainlink 1.13.0
Sensor	MFrontier
RFID	RFID high frequency
QR code	QR version 40

## Data Availability

Relevant codes can be obtained by contacting the first author.
